# Robotic Therapy: Cost, Accuracy, and Times. New Challenges in the Neonatal Intensive Care Unit

**DOI:** 10.3389/fphar.2019.01431

**Published:** 2019-11-26

**Authors:** Ilaria Amodeo, Nicola Pesenti, Genny Raffaeli, Gabriele Sorrentino, Alessia Zorz, Silvia Traina, Silvia Magnani, Maria Teresa Russo, Salvatore Muscolo, Laura Plevani, Fabio Mosca, Giacomo Cavallaro

**Affiliations:** ^1^Neonatal Intensive Care Unit, Fondazione IRCCS Ca’ Granda Ospedale Maggiore Policlinico, Milan, Italy; ^2^Department of Clinical Sciences and Community Health, University of Milan, Milan, Italy; ^3^Department of Statistics and Quantitative Methods, Division of Biostatistics, Epidemiology and Public Health, University of Milano-Bicocca, Milan, Italy

**Keywords:** robotic therapy, newborn, safety therapy, patient safety, robotic cost, time, accuracy

## Abstract

**Background:** The medication process in the Neonatal Intensive Care Unit (NICU), can be challenging in terms of costs, time, and the risk of errors. Newborns, especially if born preterm, are more vulnerable to medication errors than adults. Recently, robotic medication compounding has reportedly improved the safety and efficiency of the therapeutic process. In this study, we analyze the advantages of using the I.V. Station^®^ system in our NICU, compared to the manual preparation of injectable drugs in terms of accuracy, cost, and time.

**Method:** An *in vitro* experimental controlled study was conducted to analyze 10 injectable powdered or liquid drugs. Accuracy was calculated within a 5% difference of the bottle weight during different stages of preparation (reconstitution, dilution, and final product). The overall cost of manual and automated preparations were calculated and compared. Descriptive statistics for each step of the process are presented as mean ± standard deviation or median (range).

**Results:** The median error observed during reconstitution, dilution, and final therapy of the drugs prepared by the I.V. Station^®^ ranged within ±5% accuracy, with narrower ranges of error compared to those prepared manually. With increasing preparations, the I.V. Station^®^ consumed less materials, reduced costs, decreased preparation time, and optimized the medication process, unlike the manual method. In the 10 drugs analyzed, the time saved from using the I.V. Station^®^ ranged from 16 s for acyclovir to 2 h 57 min for teicoplanin, and cost savings varied from 8% for ampicillin to 66% for teicoplanin. These advantages are also capable of continually improving as the total amount of final product increases.

**Conclusions:** The I.V. Station^®^ improved the therapeutic process in our NICU. The benefits included increased precision in drug preparation, improved safety, lowered cost, and saved time. These advantages are particularly important in areas such as the NICU, where the I.V. Station^®^ could improve the delivery of the high complexity of care and a large amount of intravenous therapy typically required. In addition, these benefits may lead to the reduction in medication errors and improve patient and family care; however, additional studies will be required to confirm this hypothesis.

## Introduction

A significant number of intravenous medications are administered in the Neonatal Intensive Care Unit (NICU) daily, and harmful medication errors are more likely to occur there compared to adult settings (Kaushal et al., 2001). Newborns are particularly vulnerable to medication errors, based on the peculiarity of the developmental pharmacotherapy (Kearns et al., 2003; Chedoe et al., 2007). Prescriptions are expressed per kilogram of body weight and require accurate calculation and multiple dilutions when administered to preterm low-birth-weight infants. Drug pharmacokinetics and pharmacodynamics change dynamically based on evolving systems and organs maturation (Tayman et al., 2011; Allegaert et al., 2013; Allegaert et al., 2014; Allegaert and Van Den Anker, 2014). The resulting patient-specific variability to drug exposure may threaten drug safety, particularly for compounds with a narrow therapeutic range (Samra et al., 2011). Lastly, most of the drugs prescribed are still off-label for neonates (Conroy et al., 1999).

Given the complexity of all these issues, the medication process can be challenging in terms of cost, time, and risk of errors, particularly for preterm newborns (Conroy et al., 1999; Chappell and Newman, 2004; Kugelman et al., 2008). In order to optimize the therapeutic process, many technologic solutions have been introduced in clinical practice in recent years, such as the use of a computerized physician order entry system (CPOE), bar-coded identification, and smart-infusion pumps (Myers et al., 1998; Bates, 2007; Vardi et al., 2007). Recently, robotic medication compounding has demonstrated an improvement in the safety and efficiency of the therapeutic process in different settings. Robotic devices have been shown to be useful in the preparation of chemotherapies, adjuvant medications, and cytotoxic drugs for adult patients (Seger et al., 2012; Nam et al., 2016; Iwamoto et al., 2017; Unluturk et al., 2018; Geersing et al., 2019). In all cases, the robot was handled by the hospital pharmacists. To date, there are no reports of robotic applications in the neonatal setting.

The I.V. Station^®^ is an automated compounding robot that prepares ready-to-administer sterile medications through a fully-automated process (Omnicell, inc. www.omnicell.com). This technology has been specifically developed for the automated individualized preparation and distribution of injectable drugs ready for use, including drugs requiring multiple dilutions (either powdered or in solution). Several studies have already demonstrated that the use of the I.V. Station^®^ reduces the rate of preparation errors and the waste of injectable drugs (Flynn et al., 1997). In this study, we compare the advantages obtained from the robot-assisted preparation of injectable drugs by the I.V. Station^®^, to the manual preparation in terms of accuracy, costs, and time in our NICU.

## Materials and Methods

### Study Design

This study was conducted in the NICU of Fondazione IRCCS Ca’ Granda Ospedale Maggiore Policlinico in Milan in 2016 for 2 months after the introduction of the I.V. Station^®^ technology. We performed an *in vitro* experimental controlled study to analyze the accuracy, cost, and preparation time of medication with robot-assistance compared to manual preparation.

The analysis included 10 injectable drugs that were either powdered or liquid. For each drug, 10 manual preparations and 10 automated preparations were compared, for a total of 200 samples (100 manual and 100 automated). Prescriptions have been made considering a hypothetical newborn patient of 1000 g of body weight. Dosages were prescribed based on the pediatric and neonatal therapeutic dosage handbook (Taketomo et al., 2018). The updated annual consumption of each drug calculated from January to December 2018 in listed in **Table 1**.

**Table 1:** Drugs, composition, therapeutic dose, and number of annual prescriptions.

*Drug*	*Composition*	*Therapeutic dose*	*Administrations/year*
Acyclovir	powder	20 mg/kg 3 times a day for 14-21 days	33
Ampicillin	powder	50 mg/kg 3 times a day for 7 days	6585
Ampicillin+Sulbactam	powder	50 mg/kg 2 times/day for 7 days	842
Amoxicillin+Clavulanic Acid	powder	50 mg/kg 2 times/day for 7 days	3010
Dobutamine	liquid	5 mcg/kg/min	243
Fluconazole	liquid	3 mg/kg every 72 hours (prophylaxis)	505
Metronidazole	liquid	7,5 mg/kg every 12-24 hours for 14 days	1136
Paracetamol	liquid	10 mg/kg every 4-8 hours	2623
Teicoplanin	powder	8 mg/kg every 24 hours	139
Vancomycin	powder	10 mg/kg every 24-72 hours for 7-21 days	6452

Samples intended for *in vitro* analyses were not utilized for clinical purposes. Since patients were not directly involved in the study, our investigation did not require ethical approval.

### Accuracy

Accuracy was calculated as <5% difference in the bottle weight during different stages of preparation (reconstitution, dilution, and final preparation) for both the manual and automated processes. This value reflects the accuracy of the concentration of the drug at the end of each step.

#### Automated-Preparation

Using the I.V. Station^®^, drugs were prepared and multiple controls were performed at different steps, specifically:

- *Reconstitution check*: for the powdered drugs (Acyclovir, Ampicillin, Ampicillin + Sulbactam, Amoxicillin + Clavulanic Acid, Teicoplanin, Vancomycin), the I.V. Station^®^ maintained accuracy within a range of ±5% by assessing the weight of the solvent injected.
- *Dilution check*: for drugs requiring dilution (Acyclovir, Ampicillin, Ampicillin + Sulbactam, Amoxicillin + Clavulanic Acid, Dobutamine, Teicoplanin, Vancomycin), the I.V. Station^®^ maintained accuracy within a range of ±5% by assessing the weight of the drug injected.
- *Final check*: for all preparations, the I.V. Station^®^ maintained accuracy within a range of ±5% by assessing the weight of the final product.

#### Manual Preparation

Drugs were prepared by 6 nurses with at least 5 years of experience working in our NICU. No specific training was conducted before starting the study since we considered a minimum of 5 years NICU expertise sufficient for the purpose of the study.

The following formula was applied as we were aware of the density of each drug:

•Accuracy%=(measured weight−ideal weight)/(ideal weight)×100

Multiple controls were performed at different steps, specifically:

- *Reconstitution check*: for powdered drugs (Acyclovir, Ampicillin, Ampicillin + Sulbactam, Amoxicillin + Clavulanic Acid, Teicoplanin, Vancomycin), the nurse maintained accuracy within a range of ±5% by assessing the weight of the solvent injected.
- *Dilution check*: for drugs requiring dilution (Acyclovir, Ampicillin, Ampicillin + Sulbactam, Amoxicillin + Clavulanic Acid, Dobutamine, Teicoplanin, Vancomycin) the nurse maintained accuracy within a range of ±5% by assessing the weight of the drug injected.
- *Final check*: for all preparations, the nurse maintained accuracy within a range of ±5% by assessing the weight of the final product.

### Costs

We calculated and compared the overall cost of the manual vs. automated drug preparation, considering a detailed list of items including bottles, syringes, needles, caps, solvents, gloves, sterile gauze, and stoppers. For manual preparations, costs were calculated based on a single dose of medication. For the IV Station^®^, since dilution takes place once, a higher number of vials are often needed to obtain the desired concentration of the stock solution, from which the IV Station^®^ obtains the different doses of medication, but also has a larger amount of final product available. Hence, the costs included all the materials used to obtain the stock solution; however, multiple administrations can be obtained.

The costs of electricity, machine maintenance, days of detention due to possible damage or machine failure over the 2 month observation period were not considered.

### Preparation Time

For each manual preparation, we considered the time required to walk to the laminar flow hood at nurse’s station, prepare a single dose of each drug, prime the intravenous line, and return to the patient to begin drug administration. For automated compounds, we considered the time required to walk to the I.V. Station^®^, withdraw all the drugs already prepared by the robot, return to the laminar flow hood to complete the preparation and priming of the intravenous line, and return to the patient to begin drug administration.

### Statistical Analysis

Descriptive statistics for each step of the process are presented as mean ± standard deviation or median (range) for both the I.V. Station^®^ and manual preparations. The cost of each drug preparation is fixed, as a result of calculations based on the value of the drug itself and the overall material required. Therefore, a statistical comparison between the cost of the I.V. Station^®^ and manual preparation cannot be conducted due to the absence of variability in the estimates. One-way ANOVA was used to compare time savings between the drugs for single preparations. Time differences between the I.V. Station^®^ and manual preparations are reported. Statistical analysis is not informative when studying differences between the I.V. Station^®^ and manual accuracy because all values lay in the range of ±5%.

Estimates of costs/savings and preparation times are presented using a heatmap plot. Boxplots are used to show the distribution of time for each drug analyzed. Statistical analyses were performed using R version 3.5.3 (R Foundation for Statistical Computing, Vienna, Austria).

## Results

### Accuracy

The data concerning the accuracy of manual and robotic preparations are depicted in **Table 2**. For the I.V. Station^®^ preparations, the median error observed during reconstitution, dilution, and final therapy ranged within ±5% accuracy. Narrow ranges of error were observed, and they were always included in the ±5% interval.

**Table 2:** The accuracy of manual and robotic preparations.

Drug	Type	Reconstitution		Dilution		Final Therapy	
		Median Error%	Range%	Median Error%	Range%	Median Error%	Range%
Acyclovir	IV	2.6	2.0; 3.8	-0,5	-1.2; 0.8	3.1	2.1; 4.0
	M	-0.3	-2.9; 2.5	-1.8	-5.5; -0.4	-1.7	-3.1; -0.5
Amoxicillin + Clavulanic Acid	IV	2.5	-0.4; 2.8	0.6	0.1; 1.9	0.3	-0.5; 1.4
	M	-0.2	-3.5; 1.5	4.0	1.0; 5.3	-1.1	-2.3; 0.1
Ampicillin	IV	2.1	1.4; 2.6	-1.4	-3.6; 1.3	3.1	2.1; 4.0
	M	-0.2	-7.7; 6.9	3.9	0.2; 4.3	2.2	-1.4; 5.3
Ampicillin + Sulbactam	IV	2.8	2.0; 3.2	-0.5	-2.8; 1.0	-0.4	-1.1; 0.9
	M	0.0	-3.6; 3.6	6.3	5.0; 7.1	1.4	-0.4; 3.5
Teicoplanin	IV	1.2	-2.4; 1.8	-1.3	-2.1; 0.8	1.0	-1.5; 4.0
	M	0.0	-2.7; 1.2	-2.4	-3.6; -1.0	0.8	-0.4; 1.5
Vancomycin	IV	0.9	-5.0; 1.8	-0.4	-4.1; 1.3	1.0	-4.7; 3.7
	M	-0.8	-4.4; 1.0	1.2	0.1; 3.4	1.8	-0.9; 2.9
Dobutamine	IV			2.5	-1.0; 3.7	-0.2	-3.1; 1.8
	M			-3.5	-4.4; -2.8	-2.1	-3.1; -1.6
Fluconazole	IV					-1.0	-3.6; 0.9
	M					2.2	-1.6; 2.8
Metronidazole	IV					-0.7	-4.6; 1.7
	M					3.2	-0.6; 5.0
Paracetamol	IV					0.4	-2.0; 3.3
	M					4.1	-1.8; 6.2

IV, I.V.Station^®^; M, manual.

**Figure 1:**
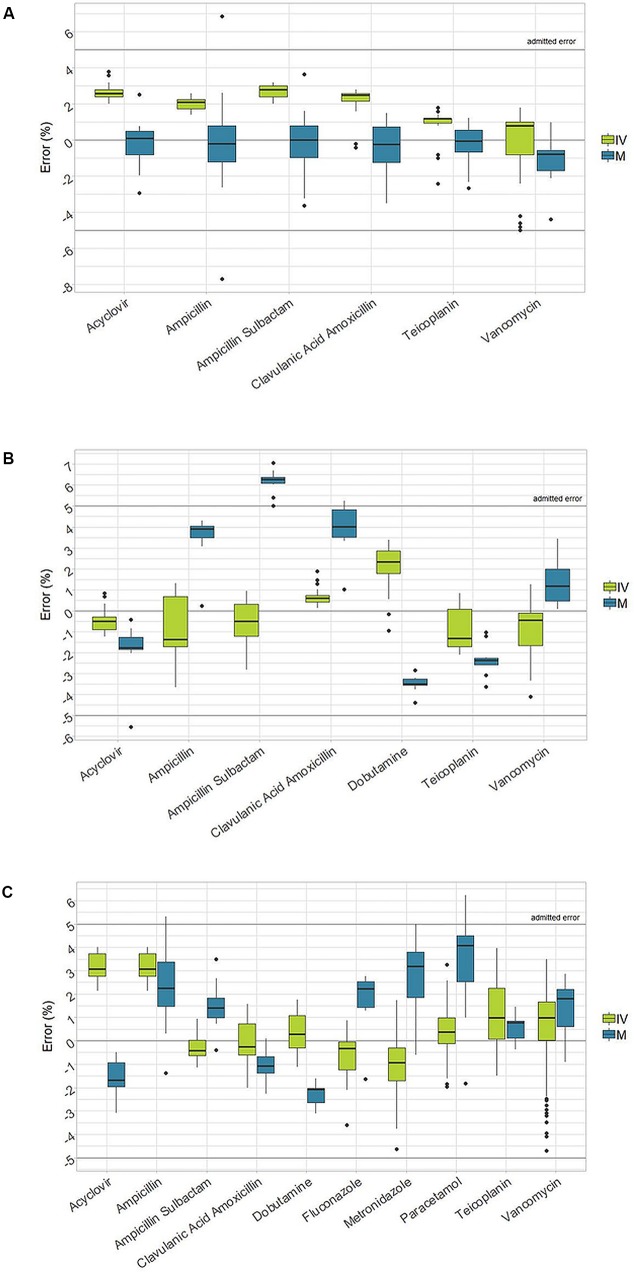
Box-plot of accuracy in reconstitution, dilution and final product between I.V. Station^®^ and manual preparations (IVS: I.V. Station^®^; M: manual). **(A)**: Accuracy in reconstitution. **(B)**: Accuracy in dilution. **(C)**: Accuracy of the final drug.

In the case of manual preparations, the median error lied in the range of ±5% accuracy, with the exception of the Ampicillin + Sulbactam, in which a median error 6.3% was observed during the dilution check. Moreover, higher variability and wider ranges of error were observed, which sometimes exceeded the ±5% limits. Indeed, in 5% of all manual preparations, the accuracy was outside the admitted interval between the range of ±5% (**Table 2**). A graphical representation of the accuracy in reconstitution, dilution, and final product for manual and robotic preparations is depicted in **Figures 1A–C**.

### Cost and Savings

**Figure 2:**
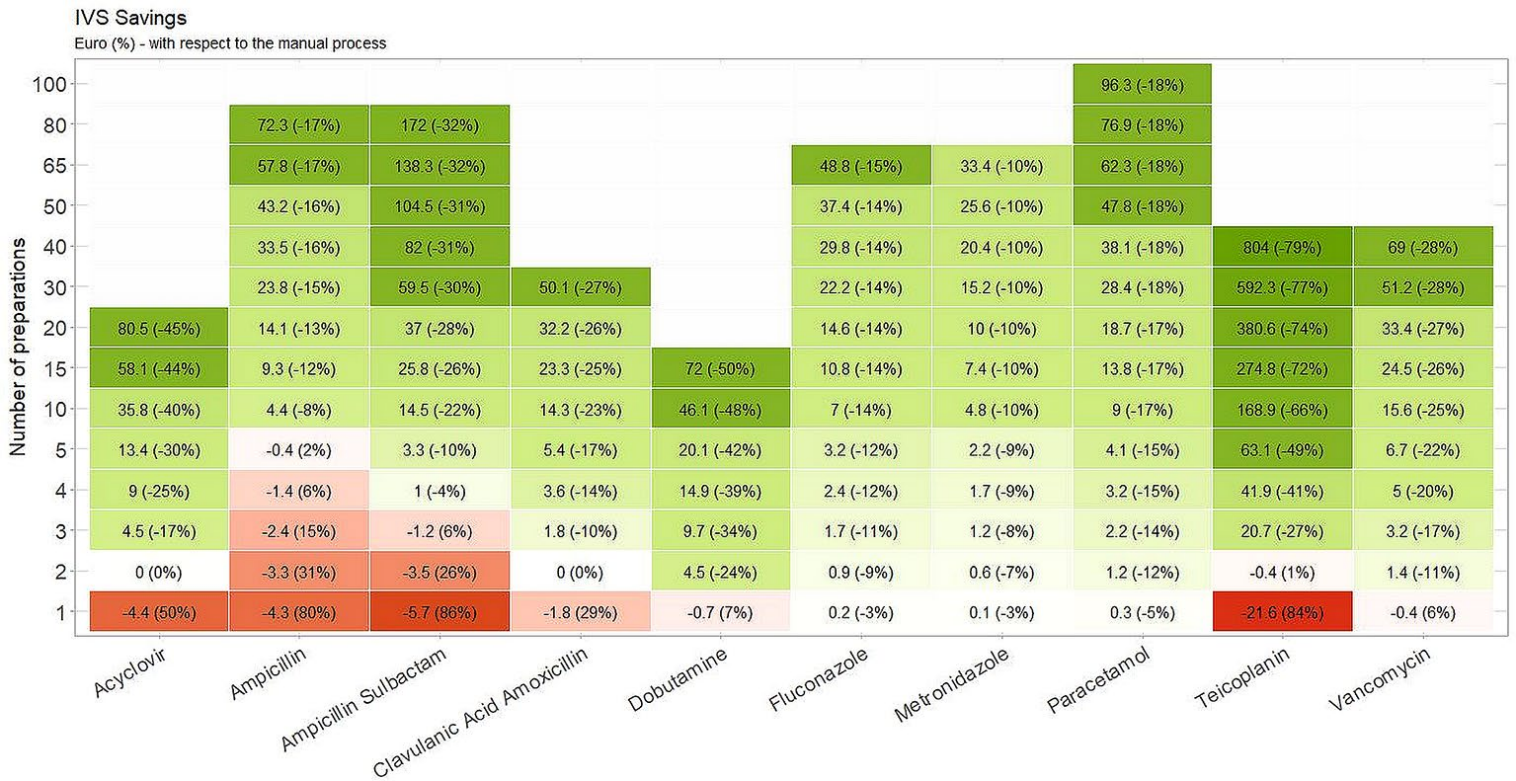
Estimated cost savings. The robotic preparations result more expensive than the manual one in the red boxes. As the amount of preparations increases, I.V. Station^®^ leads to an optimization of materials consumption and costs, becoming equal to manual preparation (white boxes) or even less expensive (green boxes).

The costs related to manual and automatic drug preparations and the number of vials needed to be prepared for a single dose of medication are listed in **Table 3**. A projection of the expected costs savings with the I.V. Station^®^ compared to manual drug preparation is displayed in **Figure 2**. For each drug, the number of drug preparations needed to amortize the robotic preparation is shown. In our study, when a low number of preparations was required, the robotic process was more expensive than the manual one (red boxes). As the number of preparations increased, the I.V. Station^®^ optimized the materials consumed and cost, and eventually equaled the cost of manual preparation (white boxes) or became even less expensive (green boxes). As shown in the heatmap, if a single preparation was considered, the manual method was more cost-effective in four out of 10 cases. However, with the 10 drug preparations analyzed, the I.V. Station^®^ led to substantial savings for all the cases considered. The expected savings ranged from 8% for ampicillin to 66% for teicoplanin and may continue to rise as the total amount of final product increased.

**Table 3:** Costs of the single vial, number of vials and relative cost for the preparation of a single dose of medication (IV Station^®^ and manual).

	IV Station										Manual									
	Acyclovir	Ampicillin	Amoxicillin + Clavulanic Acid	Ampicillin + Sulbactam	Dobutamine	Fluconazole	Metronidazole	Paracetamol	Teicoplanin	Vancomycin	Acyclovir	Ampicillin	Amoxicillin + Clavulanic Acid	Ampicillin + Sulbactam	Dobutamine	Fluconazole	Metronidazole	Paracetamol	Teicoplanin	Vancomycin
Single vial cost (€)	7.70	4.08	2,42	6.80	4.69	0.50	0.29	0.60	41.60	1.04	7.70	4.08	2.42	6.80	4.69	0.50	0.29	0.60	41.60	1.04
Number of vials (n)	2	5	2	5	1	1	1	1	2	1	1	1	1	1	1	1	1	1	1	1
Total cost of the drug (€)	15.40	20.40	4.84	34.00	4.69	0.50	0.29	0.60	83.20	1.04	7.70	4.08	2.42	6.80	4.69	0.50	0.29	0.60	41.60	1.04
Vial drug content (mg)	250	1000	1200	1000	250	100	500	1000	200	500	250	1000	1200	1000	250	100	500	1000	200	500
Vial drug volume (mL)	0	0	0	0	20	50	100	100	0	0	0	0	0	0	20	50	100	100	0	0
Mean drug doses pro Kg (mg/Kg/dosis)	20	50	50	50	5	3	7.5	10	8	10	20	50	50	50	5	3	7.5	10	8	10
Volume of Drug Reconstitution (mL)	5	5	5	5	0	0	0	0	5	5	10	20	20	20	30	0	0	0	20	100
Final dilution volume (mL)	108	120	108	120	109	50	100	100	108	104	50	20	20	20	50	50	100	100	20	100
Final drug concentration (mg/ml)	3.7037	33.3333	14.8148	33.3333	1.0321	2	5	10	2.9630	3.8462	5	50	60	50	5	2	5	10	10	5
Administration volume (mL)	5.4	1.5	3,4	1.5	7.0	1.5	1.5	1.0	2.7	2.6	4.0	1.0	1.0	1.0		1.5	1.5	1.0	0.8	2.0
Drug stability (h)	24	12	1	12	48	48	48	48	48	48	ns	ns	ns	ns	ns	ns	ns	ns	ns	ns
Number of possible administrations (n)	20	80	32	80	16	33	67	100	40	40	1	1	1	1	1	1	1	1	1	1
Num. admin. amortize costs (n)	2	3	2	4	2	1	2	2	2	2	1	1	1	1	1	1	1	1	1	1

h, hours; mg, milligrams; mL, milliliters; n, numbers; ns, not store. Doses of amoxicillin + clavulanate and ampicillin + sulbactam are referred to as amoxicillin and ampicillin alone.

### Preparation Time

**Figure 3:**
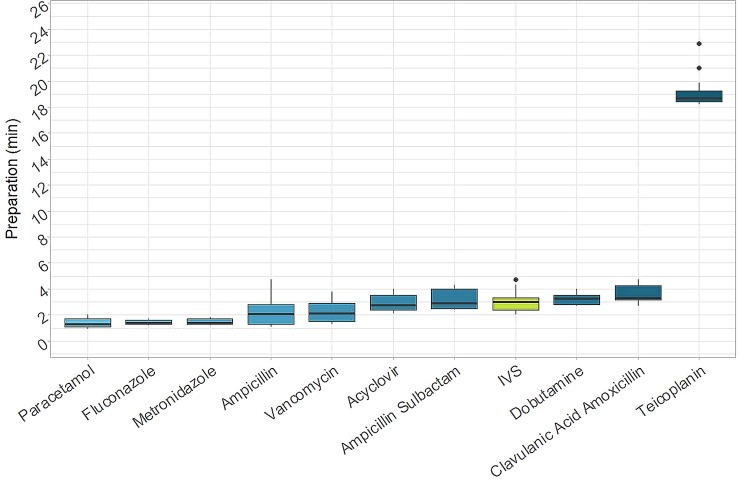
Times for preparations. Time needed for the preparation of each drug separately (IVS: I.V. Station^®^).

The time needed to prepare each drug separately is shown in **Figure 3**. In almost all cases, the time required to prepare a single manual dose was less than 4 min and ranged from 1 min 17 s for paracetamol to 3 min 18 s for Amoxicillin + Clavulanic Acid. The only exception was with teicoplanin, in which a single preparation took up to 18 min 43 s. In this case, the relevant difference was due to the difficulty associated with manual reconstitution and dilution of the powdered drug.

The median time required for a single I.V. Station^®^ preparation was 2 min 59 s. This was in line with a majority of the manual results, although it was higher compared to paracetamol, fluconazole, and metronidazole (*p*-value < 0.001) and lower than amoxicillin + clavulanic acid (*p*-value = 0.007) and teicoplanin (*p*-value < 0.001).

**Figure 4:**
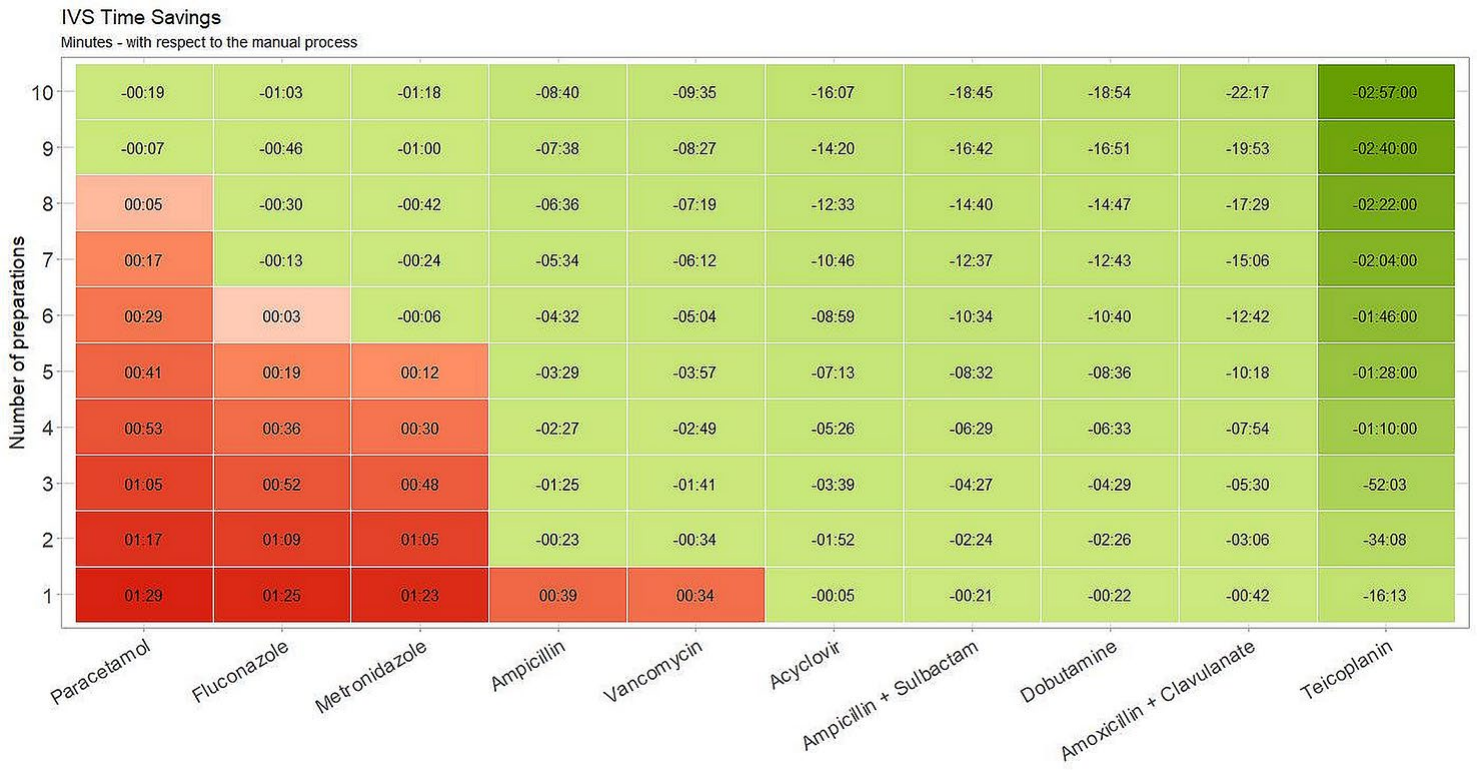
Estimated time savings. I.V. Station^®^ expected time-saving compared to manual preparation. With a low number of doses, I.V. Station^®^ could result in more time-consuming than the manual method (red boxes). With the increase of preparations, I.V. Station^®^ leads to a progressive optimization of the preparation process (green boxes).

**Figure 4** shows a projection of the expected time-savings associated with the I.V. Station^®^ compared to manual preparation. For each drug, the number of administrations required to amortize the I.V. Station^®^ preparation times are depicted. In our study, when a low number of doses were required, the I.V. Station^®^ was more time-consuming than the manual method (red boxes) in some instances. For example, if we required a single preparation, the I.V. Station^®^ was more expensive than the manual method in half of the cases analyzed. As the number of preparations increased, the I.V. Station^®^ led to a progressive optimization of the preparation process (green boxes). The I.V. Station^®^ led to substantial time-savings in all the cases analyzed. The time saved ranged from 16 s for acyclovir to 2 h and 57 min for teicoplanin and may continue to rise even as the total number of medications prepared increase.

## Discussion

Medication errors are defined as any preventable event that can lead to inappropriate medication use or patient harm. Errors can occur at any stage in the medication-use process (prescribing, transcribing, dispensing, administering, monitoring) (Aronson, 2009). The therapeutic process includes several stages; each of which are potentially at risk for medication errors (Aronson, 2009). Infants who require more intensive levels of care are at a higher risk for medication errors and potentially fatal errors are three times more likely to occur in the NICU than in adult wards (Kaushal et al., 2001). Prescribing and drug administration challenges place newborns at risk of 10-fold and up to 100-fold potentially fatal dosing errors (Chappell and Newman, 2004; Taheri et al., 2013).

Preterm babies require a more intensive level of care and more complex therapy, which exposes them to a higher risk of iatrogenic events. Thus, the incidence of medication errors that occur during the care of extremely preterm newborns is reported to be quite high near 57%, compared to 3% when caring for full-term infants (Kugelman et al., 2008). In this patient category, adequate drug dosing requires serial dilutions and manipulation of the solutions, thus increasing the odds of an incorrect dose, concentration, and contamination of the final solution (Kugelman et al., 2008). As previously demonstrated in the literature, manually-prepared drugs in the ICU setting frequently show significant deviations from the target concentration, while automated-prepared drugs show less variability (Allen et al., 1995; Parshuram et al., 2003; Wheeler et al., 2008; Dehmel et al., 2011; Seger et al., 2012). This wide variability can lead to harmful consequences, such as adverse reactions due to overdosing or loss of efficacy due to underdosing (Kugelman et al., 2008). In a recent study, Iwamoto et al. demonstrated that the robotic preparation of antineoplastic drugs using APOTECA-chemo had higher accuracy and a lower absolute dose error compared to manual preparation. The risk of overdose significantly reduced, resulting in safer cancer treatment (Iwamoto et al., 2017). Geersing et al. also demonstrated that APOTECA-chemo preparations were microbiologically safe (Geersing et al., 2019).

Our data confirm that the I.V. Station^®^ can reduce variability and thus improve accuracy at any step of the medication process (**Table 2**, **Figure 1**). Although manual method results in a tolerable median error, it shows wider variability among different preparations, with some dropping outside of the ±5% admitted interval. As recently reported in the literature, ward-based manually prepared solutions can deviate in concentration conformity more often than machine-made solutions (Kugelman et al., 2008). As can be expected in any operator-dependent process, the accuracy of the final therapy could not be guaranteed, without the possibility to identify and discard those preparations which do not respect the desired concentration. A centralized, automated preparation of standardized solutions has already been proposed as an effective means to reduce preparation error in everyday practice (Kugelman et al., 2008).

Unlike manual preparations, the I.V. Station^®^ is set to automatically discard preparations that do not respect the predetermined range of accuracy. Hence, our data are consistent with this hypothesis. For this reason, the margin of error observed with the I.V. Station^®^ never dropped outside the ±5% accuracy interval in any of the steps analyzed (reconstitution, dilution, final product). Therefore, the I.V. Station^®^ may guarantee a high level of concentration conformity, and thus increased drug safety. Moreover, when combined with other strategies (i.e., electronic medical record, computerized order entry, and bar-code system) robotic technology is expected to reduce the risk of prescription and administration errors, improving safety and workflow efficiency (Dehmel et al., 2011).

Robotic preparation appears to be safer not only for the patient but also for the staff. Seger et al. observed a significant reduction in potentially harmful staff events after the introduction of robotic preparation of an antineoplastic drug (Seger et al., 2012). Although we did not evaluate staff events in relation to the therapeutic process, we could speculate that advantages similar to those reported by Seger et al. would occur in our NICU, where work-related risk is high. Robotic technology offers the opportunity not only for safer but also for a more cost-effective medication process. Moreover, Seger et al. found out that by introducing robotic preparation of an antineoplastic drug and adjuvant medications, they considerably reduced ancillary costs associated with several components. The savings accounted for 60% of the overall cost, and when annualized for the number of antineoplastics prepared in a year, they would have saved $115,500 in material costs (Seger et al., 2012).

Our data confirm that robotic technology reduces the cost administering most drugs, especially when multiple preparations are needed. Benefits are expected to be even more remarkable when considering the huge number of medications prescribed in the NICU, since the more preparations of the same drug that are required within short period, the greater are the advantage, as the robot may use the same vial.

As shown in **Figure 2**, if the number of doses prepared through the I.V. Station^®^ is low, the material consumption is high in a first step but could be subsequently amortized by an increase in the number of preparations. The greater is the number of patients requiring the same therapy in a given period, the more remarkable the advantage. Therefore, the I.V. Station^®^ leads to greater savings in the long run, provided that the final product is consumed within the time frame in which the diluted solution remains stable.

Hence, the robotic process could be further optimized by consistently using the I.V. Station^®^, to expand production to other departments of the same hospital or other NICUs in the territory, building a distribution network with centralized production. Furthermore, automated-preparation of medications allow nurses to save time during the therapeutic process. Although manual preparation is rapid for some drugs (i.e., paracetamol, which is ready for use), some drugs require multiple dilutions and a significant amount of time to be prepared (i.e., teicoplanin) (**Figure 3**).

The I.V. Station^®^ prepares multiple drugs in a sequence that can be withdrawn at once (**Figure 4**), thus inducing relevant time savings compared to manual preparation. For liquid drugs that are ready to be used (as paracetamol), the advantage commences when the number of doses to be prepared is more than nine. In analyzing the other compounds, savings begin with a lower number of preparations and is highest with teicoplanin, as robotic process saves a great deal of time from the first administration. With costs savings, the more intravenous therapy required, the greater the advantage in terms of procedural efficiency. The saved “drug preparation time” can, therefore, be used for the direct care of the neonate. While the robot is working, the nurse could remain at the patient’s bedside to better assist the baby wherever necessary, engage and educate the family, in an effort to provide the best care possible. As a result, the bond between the newborn and parents can be strongly enhanced. All these positive effects are not currently quantifiable but could represent a strong point in favor of smart robotics in the NICU setting.

Our study has some limitations. We analyzed a small number of injectable drugs, which only represents a small proportion of the intravenous therapy administered in the NICU. However, we have included the compounds that are the most commonly prescribed by neonatologists. Our analysis did not include costs concerning electricity, machine maintenance, or days of detention due to possible damage or failure of the robot. However, in 2018, the inactivity rate of the I.V. Station^®^ was almost negligible (2.5% = 9.2/365 days), with a minimum time lag of 4.8 min up to 2.5 days of the stop. Seger et al. found some mechanical or software failure events associated with robotic preparations, which did not have harmful consequences on the patients but affected workflow efficiency and wasting of some medication (Seger et al., 2012). These are important limitations of robotic technology. The impact of ancillary costs, robot, or software failure, must be further characterized, and strategies for avoiding waste need to be implemented. Another limitation is that analysis of the microbiological safety of robotic compounds was not analyzed in this study. However, a preliminary microbiological analysis confirming the bacteriological safety was performed in 2013 before the implementation of the robot in our Unit. Based on the standard operating procedures of the I.V. Station^®^, the sterility of drug preparations is guaranteed for 24 h.

Based on our on-site microbiological surveillance, we have extended the sterility for liquid preparations for up to 72 h. Over 1 year, we analyzed microbiological cultures taken both from pharmaceutical preparations and the surface of the robot (daily during the first month, then weekly for three months and monthly for the rest of the year), which turned out to be negative (unpublished data). Lastly, the observation period was limited to 2 months after the introduction of the robot. To better define the advantages of the I.V. Station^®^ in clinical practice, further analysis must be conducted on a greater number of compounds, and during a more extended period.

## Conclusions

Our data demonstrate that the I.V. Station^®^ may support the therapeutic process in the NICU. Benefits are related to accuracy in drug preparation, cost, and time-saving. These advantages are particularly important in the NICU, where the I.V. Station^®^ could facilitate the high complexity of care, nursing workload, and the significant amount of intravenous therapy typically administered. Robotics may positively impact the patients and their families, by allocating the human resources (i.e., nurses’ time and effort) to neonatal care. A possible reduction in medication errors due to the introduction of automated procedures is also possible: however, additional studies are required to confirm this hypothesis. Efforts should be directed at drugs that are not currently available in vials, in order to extend the number of pharmaceutical drugs that can be prepared by the robot.

## Data Availability Statement

The datasets generated for this study are available on request to the corresponding author.

## Author Contributions

GC, GR, GS, LP, and FM contributed conception and design of the study. GS, AZ, ST, SMa, MR, and SMu performed the experiments. IA, GC, GR, and NP wrote the first draft of the manuscript. All authors contributed to manuscript critical revision, read and approved the submitted version.

## Disclaimer

None of the authors of the manuscript hold the copyright of I.V. Station. The robot was donated by the Vodafone Foundation, after a public money collection, to AISTMAR Onlus (https://www.aistmar.it/), a charity association, that donated it to NICU of Fondazione IRCCS Ca’ Granda Ospedale Maggiore Policlinico, Milan in 2013.

Our video showing the robot working in the Unit is available on the website: https://www.youtube.com/watch?v=ATDlPD-1j_Q&t=10s. Furthermore, the robot manufacturer is aware of our willingness to carry out a comparative study even in the absence of funding, as the study was no profit. Therefore, being a product owned by NICU, we confirm that we have the correct authorizations to evaluate this product.

## Conflict of Interest

The authors declare that the research was conducted in the absence of any commercial or financial relationships that could be construed as a potential conflict of interest.
